# Cancer therapy by resuscitating Notch immune surveillance

**DOI:** 10.1186/2051-1426-2-S1-O1

**Published:** 2014-02-24

**Authors:** Anil Shanker, Duafalia F Dudimah, Samuel T Pellom, Roman V Uzhachenko, David P Carbone, Mikhail M Dikov

**Affiliations:** 1 Department of Biochemistry and Cancer Biology, School of Medicine, Meharry Medical College, Nashville, TN, USA; 2 Host-Tumor Interactions Program, Vanderbilt-Ingram Comprehensive Cancer Center, Vanderbilt University, Nashville, TN, USA; 3 School of Graduate Studies and Research, Meharry Medical College, Nashville, TN, USA; 4 Department of Medicine, James Cancer Center, The Ohio State University, Columbus, OH, USA

## 

The immunosuppressive tumor microenvironment perturbs numerous immune regulatory networks and usurps host antitumor immunity. We discovered that tumor interferes with host hematopoietic Notch system in lung cancer patients [1]. The resultant decrease in immune Notch signaling could be a major causative link in the inadequate induction of antitumor immunity. Interestingly, administration of the novel Delta-like ligand 1 (DLL1) multivalent cluster [1] and the FDA-approved proteasome inhibitor drug bortezomib, which also sensitizes tumors to death signals [2,3], restored the tumor-induced decrease in immune Notch. Bortezomib increased the expression of Notch target genes Hes1 and Hey1 in thymus, lymph node, and spleen of tumor-bearing mice. Moreover, bortezomib administration decreased the proportion of regulatory T cells and enhanced antitumor T cell production of IFN-γ. Results indicate that bortezomib-induced activation of Notch target genes Hes1 and Hey1 is through its inhibition of NFκB while its activation of Deltex1 is mediated via PI3K. The potential of modulating antitumor Notch signaling by the prototypic DLL1 cluster in combination with bortezomib presents exciting opportunities to uncover multi-pronged immune stimulatory regimens. Therapeutic restoration of immune Notch signaling by bortezomib could provide effective treatment and recurrence-free survival in cancer patients by breaking tumor resistance, enhancing immune surveillance, and sustaining robust anti-tumor immunity.
